# Challenges and opportunities in testing sensorimotor processing with tendon vibration and transcranial magnetic stimulation in subacromial impingement syndrome: A case series

**DOI:** 10.1371/journal.pone.0305545

**Published:** 2024-07-11

**Authors:** Émilie Bouchard, Lydiane Lauzier, Mathieu Boudier-Revéret, Laurence Munger, Kossi Épiphane Ketounou, Marie-Pier Perron, Suzy Ngomo, Stéphane Sobczak, Louis-David Beaulieu

**Affiliations:** 1 Laboratoire BioNR, Centre Intersectoriel en Santé Durable, Université du Québec à Chicoutimi, Saguenay, QC, Canada; 2 Physical Medicine and Rehabilitation Service, Department of Medicine, Centre hospitalier de l’Université de Montréal, Montréal, QC, Canada; 3 Département d’anatomie, Université du Québec à Trois-Rivières, Trois-Rivières, QC, Canada; 4 Chaire de Recherche en Anatomie Fonctionnelle, Université du Québec à Trois-Rivières, Trois-Rivières, QC, Canada; Harvard Medical School, UNITED STATES

## Abstract

**Background:**

Non-invasive neurostimulation like muscle tendon vibration (VIB) and transcranial magnetic stimulation (TMS) can provide valuable insights on mechanisms underlying sensorimotor dysfunctions. However, their feasibility in the context of painful musculoskeletal disorders like shoulder impingement syndrome (SIS) remain uncertain.

**Methods:**

The present work used a case series design including 15 participants with SIS, as well as a secondary group-based analysis comparing participants with SIS to 15 healthy counterparts. Proprioceptive processing was tested by VIB-induced kinesthetic illusions of shoulder abduction, and TMS tested corticospinal excitability of the upper trapezius. Detailed individual data were collected, including any technical challenges and feasibility issues encountered.

**Results:**

VIB was in general well-tolerated and elicited a perceptible kinesthetic illusion in 13 participants with SIS and 14 controls. TMS presented with several challenges related to discomfort, fear-related behaviors, technical problems and high motor thresholds, especially in participants with SIS. It was only possible to collect all TMS measures in 5 participants with SIS (for both the painful and non/less-painful sides), in 7 controls on their dominant side and 10 controls on the non-dominant side. The only significant group-based analysis was a lower illusion speed/amplitude on the painful versus non-painful side in persons with SIS (p = 0.035).

**Conclusion:**

Our study provides preliminary data on challenges encountered with TMS and VIB of trunk/proximal muscle in persons with SIS and healthy counterparts. It might help future studies to better address those challenges beforehand and improve the overall feasibility and impact of neurostimulation tools in musculoskeletal disorders.

## Introduction

The lifetime prevalence of suffering from musculoskeletal shoulder disorders like subacromial impingement syndrome (SIS) varies from 6.7% to 66.7% of the population [1]. In 50% of cases symptoms such as pain, stiffness and muscle weakness will persist for more than one year, causing long-term personal and socio-economic consequences [1]. Although the etiology of SIS remains unclear [2], scapular dyskinesia, resulting from weakness and a lack of coordination of scapular muscles during shoulder elevation (upper and lower trapezius, serratus anterior) [3, 4] is often observed in patients with shoulder disorders [3–6]. An altered shoulder proprioception (i.e. internal sense of joint and body movement and position in space) has been recently proposed as another contributing factor to the lack of control over scapular muscles during shoulder elevation [7, 8]. These sensorimotor impairments could be in turn exacerbated by the negative impact of pain itself on sensory and motor functions [9, 10].

However, the neural mechanisms involved in these sensorimotor alterations remain understudied. Non-invasive neurostimulation devices offer new opportunities to investigate specific mechanisms involved in the processing of proprioceptive afferents and cortico-motor control of scapular muscles. Muscle-tendon vibration (VIB) is known as an effective method for depolarizing muscle spindle afferents, i.e. somatosensory receptors playing a central role in proprioception [11]. In the absence of visual and cutaneous feedbacks, VIB elicits kinesthetic illusions coherent with the stretching of the vibrated muscle. So far, VIB has been used to investigate mechanisms of sensorimotor processing in healthy individuals [12–14], and more recently to identify and treat proprioceptive deficits in different populations such as chronic stroke [15, 16]. In conditions affecting shoulder control, persons with scapulohumeral muscular dystrophy showed a preserved ability to perceive kinesthetic illusions and responded positively to a VIB-based treatment with improved shoulder range of motion and self-rated health [17, 18]. To date, no study verified if persons with SIS present an altered perception of VIB-induced illusions. VIB could represent a useful tool for the clinical management of SIS, by providing a sensory-based method for evaluating and treating neural functions involved in the processing of key afferents subserving motor control [19, 20].

Apart from VIB, transcranial magnetic stimulation (TMS) of the primary motor cortex (M1) provides a non-invasive and painless method to test the integrity and excitability of the corticospinal system, which is the main system for voluntary control of skeletal muscles [21]. TMS can also be used to track changes within the corticospinal system resulting from pathological conditions or in response to therapies targeting sensorimotor deficits [22]. Two study used TMS with the SIS population and highlighted a lower corticospinal excitability for the control of scapular and deltoid muscles compared to healthy controls [23, 24].

Despite preliminary evidence supporting the value of both TMS and VIB for investigating the integrity of sensorimotor control mechanisms in shoulder disorders, their feasibility has not been extensively described. This knowledge would provide valuable inputs for future investigations, especially considering how challenging neurostimulation can be when targeting trunk and proximal muscles. Therefore, the primary objective of this case series was to describe how persons with SIS respond to TMS and VIB-related procedures, as well as feasibility issues encountered. A secondary objective was to verify whether TMS and VIB outcome measures can discriminate between persons with SIS and healthy counterparts, and between the painful and non-painful sides in SIS group. The hypothesis was that TMS and VIB procedures will be mostly challenged by discomfort in persons with SIS, but results will show lower clearness and speed/amplitude of VIB-induced illusions, along with lower corticospinal excitability in the painful shoulder vs. the non-painful one and vs. healthy counterparts.

## Materials and methods

### Participants and study design

Fifteen participants with shoulder pain (5 men, 10 women, mean age 43 ± 15 years) and fifteen healthy counterparts (8 men, 7 women, mean age 46 ± 14 years; Table 1) were recruited. An *a priori* sample size for the secondary group-based analysis was estimated with the G*Power software (version 3.1.9.6 [25]). Based on VIB and TMS results from studies similar to ours [17, 18, 24], the minimal sample size to obtain a contrast between a group with shoulder disorders and healthy counterparts with a power of 95% and alpha error of 5% was 13 participants per group for VIB and 15 participants per group for TMS outcomes. The inclusion criteria for controls were: 18 years or older, in general good health with no history or present shoulder pain/injury. The group with shoulder pain needed to be diagnosed with the 3 out of 5 positive SIS tests (painful arc, Jobe test, Neer test, Hawkins-Kennedy test and resisted external rotation) [26] and having pain for more than 3 months. Exclusion criteria for both groups were cognitive disorders, any other problem affecting the shoulders, previous shoulder or cervical surgery, total shoulder prothesis, and muscles, tendons tear or capsulitis. Exclusion criteria related to TMS included pregnancy, cranial metallic or electronic implants, history of cerebral tumours or infection, history of syncope or epilepsy [27]. All participants gave their written inform consent before the experiment, which was approved by the local ethics committee (#2021–698). Furthermore, ultrasound images of subacromial shoulder structures (supraspinatus, infraspinatus, long head of the biceps brachii) were taken and verified by an expert physiatrist who helped to verify the integrity of subacromial and surrounding structures but was not an inclusion or exclusion criteria.

**Table 1:** Detailed characteristics of participants with SIS.

Case # (age/gender)	Weight (kg)	Height (cm)	Handed-ness	Painful shoulder	QuickDASH (/100)	Pain severity	GPAQ (MET-min)	Notthing-ham (/3)	Ultrasound Painful side	Ultrasound Non/less-painful side
**1** (47/F)	70.29	1.70	L	R	31.8	Moderate	7080	3	NAD	SASD slightly thickened, mild SS and IS tendinosis,
**2** (35/M)	80.73	1.73	R	R	29.1	Mild	1440	3	SASD thickened, normal limit	NAD
**3** (66/F)	77.10	1.60	R	L	43.2	Mild	0	3	Signs of enthesopathy, possibility of a SS tear, mild to moderate IS tendinosis	Signs of chronic enthesopathy, mild IS tendinosis.
**4** (43/F)	99.77	1.70	R	L	34.1	Moderate	0	3	Possibility of a small calcification	NAD
**5** (28/F)	68.03	1.30	R	L	50.0	Severe	1920	3	SASD slightly thickened	Suspicion of a SS tear <25%, mild bursitis
**6** (39/M)	104.31	1.85	R	Bilat	50.0	Moderate	4800	3	NAD	NAD
**7** (60/M)	88.44	1.72	R	L	27.3	Moderate	5760	3	Chronical SASD without effusion	Possibility of complete SS tear, possibility of tendinosis of the LHB
**8** (38/M)	113.38	1.77	R	L	20.5	Moderate	720	3	NAD	NAD
**9** (52/F)	68.03	1.63	L	R	61.4	Severe	0	3	NAD	NAD
**10** (37/M)	92.97	1.67	R	R	6.8	Mild	13480	3	Mild SS tendinosis	IS tendinosis, SASD thickened with small effusion
**11** (60/M)	96.60	1.85	R	R	36.4	Moderate	21840	3	Chronical SASD thickened, dislocation of LHB, mild IS tendinosis	NAD
**12** (43/F)	72.56	1.74	R	Bilat	27.3	Moderate	700	3	Mild chronic calcific enthesopathy,	NAD
**13** (43/F)	74.83	1.65	R	L	36.4	Moderate	4740	3	NAD	NAD
**14** (71/M)	82.54	1.72	L	R	50.0	Moderate	7200	3	Possibility of articular tear, moderate tendinosis, calcific tendinopathy, degenerative rearrangement around the small and large tuberosity,	Possibility of SS tear (SS pathology), enthesopathy, mild to moderate IS tendinosis
**15** (21/M)	77.01	1.89	R	R	18.2	Mild	6480	3	NAD	NAD

SIS = shoulder impingement syndrome; QuickDASH = Quick Disabilities of Arm, Shoulder and Hand; GPAQ = Global Physical Activity Questionnaire; MET = metabolic equivalent; NAD = no abnormality detected; SASD = subacromial-subdeltoid bursa; SS = supraspinatus; IS = infraspinatus; LHB = long head of the biceps

The study consisted of one session lasting approximately 1.5 hours. The first part included SIS tests, questionnaires, and ultrasound imagery. Then, vibration-induced kinesthetic illusion and TMS measures of corticospinal excitability of the upper trapezius muscles were obtained. The upper trapezius was chosen because: (i) it presents an altered muscle activation during shoulder elevation in SIS population [5]; (ii) it is easily accessible for surface electromyography (required for TMS measurements); (iii) the evidence related to TMS with this muscle in SIS remains debated [24].

### Participants’ characteristics

A questionnaire about shoulder pain history and symptomatology, comorbidities and sociodemographic information was first completed. Then, the level of pain and disability were established with the French-Canadian version of the Quick Disabilities of the Arm, Shoulder and Hand (QuickDASH) questionnaire [28]. The usual level of physical activity was measured using the French version of the Global physical activity questionnaire (GPAQ). Shoulder proprioception was tested with the proprioception subscale of the Nottingham Sensory Assessment [29].

### Vibration-induced kinesthetic illusions

**Fig 1:**
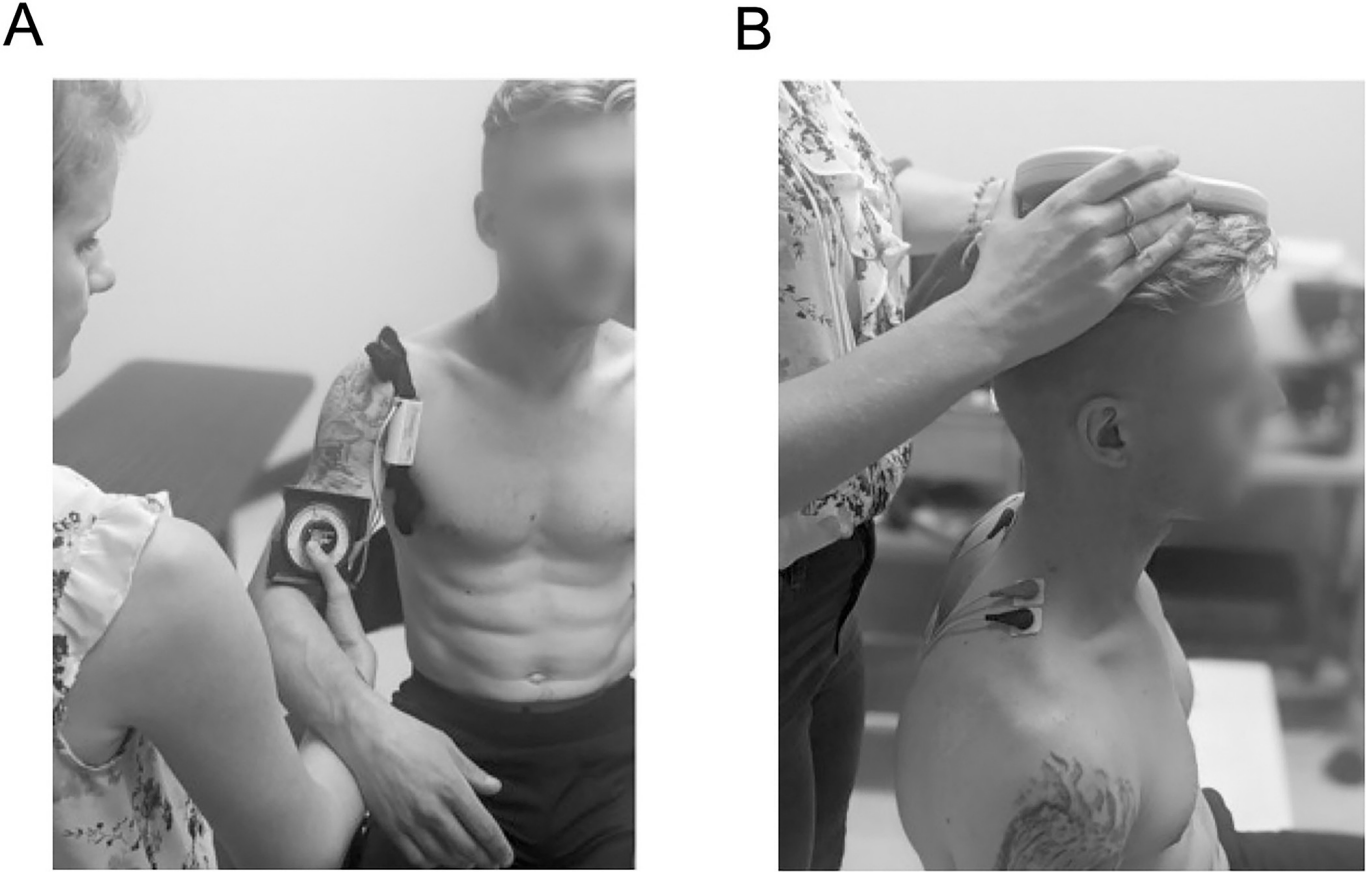
Participant’s positioning for vibration-induced kinesthetic illusions (A) and transcranial magnetic stimulation (B). Reprinted from [Bouchard Émilie. (2023). *Investiguer l’intégrité des mécanismes d’intégration sensori-motrice via la vibration tendineuse et la stimulation magnétique transcrânienne en présence d’un syndrome d’abutement*. Mémoire de maîtrise, Université du Québec à Chicoutimi] under a CC BY license, with permission from [Émilie Bouchard], original copyright [2023].

Kinesthetic illusions were elicited following guidelines from the Standardized Kinesthetic Illusion Procedure (SKIP–detailed in [30]). Participants were comfortably seated on a chair with their eyes closed, the tested arm at rest in the evaluator’s hands, leg joints around 90° and feet on the ground. Two tendon vibrators (VB115, Techno Concept, France) were placed under the armpit over tendons of shoulder adductor muscles including subscapularis, pectoralis major and latissimus dorsi to simulate a stretch of these muscles and therefore induce an illusion of shoulder abduction. The optimal joint position eliciting the most clear and precise illusions was identified and kept constant throughout vibration trials using an inclinometer (Johnson Pitch and Angle Locator, USA) attached with an elastic band on the arm (Fig 1A). Vibration was administered at 80 Hz and 1 mm during 10 s [30]. SKIP rating sheet was used to measure kinesthetic illusions in terms of clearness/precision (perfectly clear and precise = 3; moderately clear/precise = 2; vague and not precise = 1; no illusion = 0) and direction (expected direction = 1; any other direction = 0). In addition, a visual analogue scale (VAS) was used to evaluate the perceived speed/amplitude of illusions [31]. These procedures were realized on both shoulders (3 trials per side) in a random order between participants. Participants were frequently asked if the setup and procedures were comfortable, and the experimenter noted any comment or observation pertaining to feasibility or technical issues.

### Transcranial magnetic stimulation

Participants were comfortably seated with the tested shoulder at rest (Fig 1B). The resting state was chosen for TMS procedures instead of asking the participants to maintain an active contraction of their shoulder muscles to avoid inducing discomfort and fatigue, especially in our recruited participants with SIS. Surface electromyography followed SENIAM recommendations for electrodes’ placement and standard skin preparation [32]. Ag-AgCl electrodes (Kendall, Cardinal Health Canada inc. Ontario, Canada) were placed 2 cm apart on the belly of each upper trapezius muscles at 50% distance on a line between C7 spinous process and the lateral aspect of the acromion. The ground electrode was placed on C7’s spinous process. Electromyographic signals were amplified before digitization at 2 kHz sampling rate (1902 & 1401 systems, Cambridge Electronic Design Limited, UK) and computer-stored for online display and offline analysis (Spike 2 software, Cambridge Electronic Design Limited, UK). The upper trapezius’ M1 representation or ‘hotspot’ was first approximated 2 cm antero-lateral from the longitudinal fissure based on the 10–20 EEG protocol [33, 34]. A 70-mm figure-of-eight coil connected to a MagStim200^2^ stimulator (The Magstim company, UK) was placed on the scalp contralateral to the targeted upper trapezius at 45° angle with the medial line [35]. To find the hotspot, the coil was moved in small steps and stimuli were delivered at varying intensities until a position that elicited highest motor evoked potentials (MEPs) in the upper trapezius with lowest intensity of stimulation was determined. The hotspot was marked on the scalp with a surgical pen and was always re-verified before each set of measurement by giving a few suprathreshold stimuli. The resting motor threshold, defined as the intensity of stimulation resulting in measurable MEPs of ≥ 50 *μV* approximately 50% of the time [36], was established with the threshold-hunting algorithm [36–38] embedded in the motor threshold assessment tool 2.0 software (MTAT 2.0, Clinicalresearcher.org, South Carolina, USA). Then, 10 stimuli were delivered at 120% rMT to obtain the mean peak-to-peak amplitude and conduction time (latency) of MEPs. In cases where no measurable MEP was elicited even at maximum stimuli intensity, procedures were stopped, and the participant was classified as ‘absence of MEP’. During all TMS measurements, electromyographic recordings were constantly monitored visually to ensure that the upper trapezius remained at rest. All these procedures were realized on both sides, in a random order between participants. The experimenter frequently verified the participant’s comfort and noted any feasibility or technical issues encountered.

### Statistical analysis

For the primary objective (case series), descriptive analyses were used for all relevant data collected for each case of SIS, including any technical and feasibility challenges. For the secondary objective (group comparisons), analyses were realized with SPSS version 26 (Armonk NY, United States) and using an alpha level below 0.05. Nonparametric analyses were chosen for all ordinal variables (SKIP scores of illusion clearness, direction and speed/amplitude, questionnaires, sociodemographic characteristics) as well as for TMS variables (rMT, MEP amplitude and latency) since they did not respect the normal distribution as shown by the Shapiro-Wilk test and visual inspection of box-and-whisker plots. The Wilcoxon signed-rank test was applied for within-group comparisons (i.e. comparing between sides) and the Mann-Whitney U-test was used for between-group comparisons (i.e. between healthy and SIS groups). The Chi-square test was used for comparing the dichotomous classification of participants between ‘absence of MEP’ and ‘presence of MEP’.

## Results

### Participants’ characteristics

Fifteen participants with SIS and fifteen healthy counterparts were recruited. Detailed characteristics of each case of SIS are presented in Table 1, and group data are described in Table 2. Nine participants had SIS on their non dominant side, four on their dominant side and two had bilateral SIS but with one side worse than the other. QuickDASH scores of upper limb disability ranged between 20–50 points for most cases (n = 12/15), with two cases presenting milder disability levels (6.8 and 18.2 points) and one having greater disabilities (61.4 points). Two participants rated their pain severity (question #9 of the QuickDASH) as severe, nine had a moderate level of pain and four scored mild pain. Weekly physical activity level as tested using the GPAQ questionnaire met the minimal recommendations from the World Health Organization (REF) of >600 MET-minutes in all but three cases which were completely sedentary (0 MET-minute). Shoulder proprioception was normal for all participants according to the Nottingham sensory assessment. Ultrasound data showed 5 cases of SIS without any structural damage on their painful side and 7 cases in which no abnormality was found on the non/less-painful side. For all other cases, ultrasound reports mostly highlighted mild to moderate tendinosis or tissue thickening observed on either side. There were a few cases of tendon tear/dislocation and bursitis. Overall, the observed structural abnormalities were located in the supraspinatus, infraspinatus, long head of the biceps and subacromial-subdeltoid bursa on both the painful and non/less-painful sides.

**Table 2:** Participants’ characteristics for SIS and control groups (mean ± SD or median [25^th^-75^th^ IQR]).

	Control group	SIS group	*p value* *
**Sex (male/female)**	5/10	8/7	*0*.*277*
**Age (year)**	46 ± 14	43 ± 25	*0*.*48*
**Height (m)**	1.69 ± 0.09	1.70 ± 0.14	*0*.*544*
**Weight (kg)**	75.40 ± 8.90	84.44 ± 14.15	*0*.*089*
**QuickDash (/100)**	2.42 ± 4.89	35.23 ± 14.83	***0*.*000***
**GPAQ (MET minutes/week)**	6541.33 ± 7347.55	5077.33 ± 5984.60	*0*.*663*
**Nottingham Sensory Assessment—proprioception subscale (/3)**	3 [3–3]	3 [3–3]	*1*.*000*

*p value in bold is statistically significant. SD = standard deviation; IQR = interquartile range; SIS = shoulder impingement syndrome; MET = metabolic equivalent.

Group data for participants with SIS and healthy counterparts are shown in Table 2. There was no significant difference in characteristics between groups except for the QuickDASH questionnaire which was significantly (p = 0.000) higher (i.e. more impaired upper limb function) in persons with SIS.

### Vibration-induced kinesthetic illusions

In general, VIB procedures were well tolerated without adverse events or discomfort for participants in both groups. Most rather reported a pleasant experience, often eliciting spontaneous smiling. The only exceptions were for case #3 who reported moderate pain (estimated by the person as around 4/10 of intensity) and case #12 who had difficulty to relax and showed fear of movement behaviors that often required rests. As shown in Table 3, 13 out of the 15 participants were able to perceive VIB-induced illusions. Case #10 was never able to perceive an illusion on both sides and case #8 did not perceive any illusion on the painful side. Also, one participant in the healthy control group failed to perceive any illusion on its dominant shoulder and the same happen to another participant on its non-dominant side. Therefore, the success rate for eliciting perceptible illusions in our study was 91.7% (55/60 shoulders tested) which fares within expected rates based on other published reports (between 87.5% to 100% success rate) [18, 39–42]. Of note, data from participants who failed to perceive a kinesthetic illusion were not removed from the analysis since SKIP and VAS scores of zero imply that no illusion was reported.

**Table 3:** Results for vibration-induced kinesthetic illusions for each case of SIS.

Case #	Optimal angle (°)		Clearness (/3)		Direction (/1)		Speed/amplitude (/100)	
	Painful	Non/less-painful	Painful	Non/less-painful	Painful	Non/less-painful	Painful	Non/less-painful
**1**	50.0	50.0	2	3	1	1	39.3	62.5
**2**	65.0	45.0	2	3	1	1	34.3	35.4
**3**	45.0	55.0	3	3	1	1	48.0	51.5
**4**	60.0	60.0	3	3	1	1	6.4	37.1
**5**	60.0	55.0	3	3	1	1	6.4	48.5
**6**	45.0	45.0	2	2	1	1	12.7	13.7
**7**	50.0	45.0	3	3	1	1	52.8	35.0
**8**	0	45.0	0	1	0	0	0	6.4
**9**	40.0	40.0	3	3	1	1	66.5	74.7
**10**	0	0	0	0	0	0	0	0
**11**	55.0	45.0	1	3	0	1	16.7	55.0
**12**	45.0	55.0	2	0	1	0	24.0	0.7
**13**	55	50	2	3	1	1	23.6	54.1
**14**	50.0	55.0	2	3	1	1	38.0	34.3
**15**	50.0	45.0	2	3	1	1	10.3	42.3

SIS = shoulder impingement syndrome

The best illusory perceptions were reported when the shoulder was positioned between 40-65° of abduction (cf. optimal angle in Table 3). Illusions were for the most part moderately to perfectly clear and precise (i.e. SKIP scores of 2 or 3 points) and in the expected direction (SKIP scores of 1 point) of shoulder abduction. Of note, perfectly clear and precise illusions were observed more frequently on the non/less-painful side (in n = 12 cases) than the painful one (in n = 5 cases). Cases #8 and #12 perceived weak and unclear illusions on their non-painful side, with scores of clearness/precision and direction varying between 0–1 point across the three trials and mean speed/amplitude below 10%.

**Fig 2:**
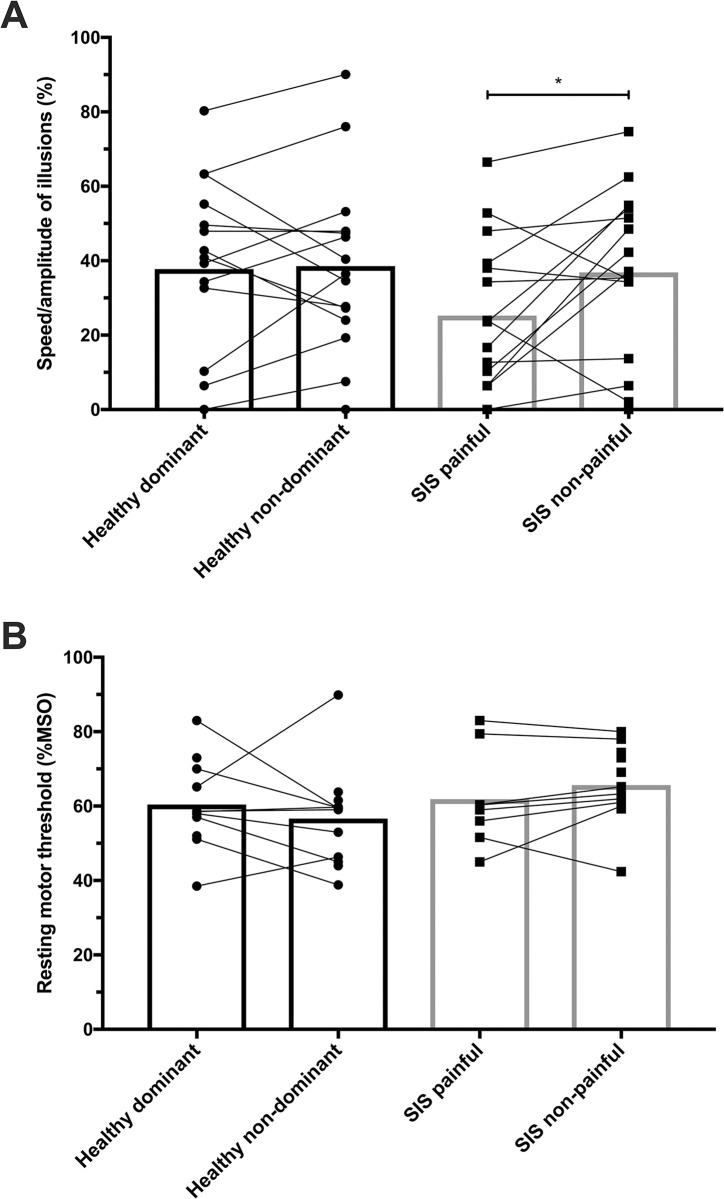
A) Individual and group (mean ± 95% confidence intervals) results for the perceived speed/amplitude of illusions rated on a visual analogue scale. SIS: shoulder impingement syndrome; * denotes a statistical (p<0.05) difference. B) Individual and group (mean ± 95% confidence intervals) results for resting motor thresholds. MSO = maximal stimulator output; SIS: shoulder impingement syndrome.

The group-based analysis failed to show significant within- or between-group difference for SKIP measures of clearness, direction and optimal joint angle, as presented in Table 4. However, illusory perceptions had significantly lower speed/amplitude when comparing the painful shoulder versus the non/less-painful one (p = 0.035). No difference was found when comparing speed/amplitude results between SIS and healthy groups. Moreover, large variations of scores were found between participants in how they rated the speed/amplitude perceived on the visual analogue scale (ranging from 0% to 90%), as shown in Fig 2A.

**Table 4:** Results of kinesthetic illusions for SIS and control groups (mean ± SD or median [25^th^-75^th^ IQR]).

	Control group			SIS group		
	Dominant shoulder	Non-dominant shoulder	*Within-group difference (p value)*	Painful shoulder	Non-painful shoulder	*Within-group difference (p value)*
**SKIP direction (/1)**	1 [1–1]	1 [0.5–1]	*0*.*564*	1 [1–1]	1 [1–1]	*1*.*000*
**Optimal joint angle (° of abduction)**	43.33 ± 18.48	46.00 ± 19.57	*0*.*372*	44.67 ± 19.32	46.00 ± 13.91	*0*.*959*
**SKIP clearness (/3)**	3 [2–3]	3 [1.5–3]	*0*.*593*	2 [2–3]	3 [3–3]	*0*.*098*
**Speed/amplitude (%)**	37.73 ± 24.38	38.54 ± 23.61	*0*.*917*	25.28 ± 20.47	36.85 ± 22.56	***0*.*035***

*p value in bold for within-group comparisons is statistically significant. No significant difference was found for between-group comparisons. SIS = subacromial impingement syndrome; SD = standard deviation; IQR = interquartile range; SKIP = Standardized Kinesthetic Illusion Procedure.

### Transcranial magnetic stimulation

Several challenges were encountered for TMS procedures. As shown in Table 5, measurable MEPs in the upper trapezius muscle and rMT identification were obtained in 8/15 and 12/15 participants with SIS, respectively for the painful and non/less-painful sides. However, MEP amplitudes and latencies at 120% rMT were not possible to collect in 10 participants on the painful and 10 participants on the non/less-painful side. We noted three main reasons for the missing data: (i) the participant refused to carry on the tests because of discomfort or fear-related behaviors toward the technology (happened in cases #3-4-5-6-7-9-12); (ii) TMS-related electrical artifact was too large for a reliable MEP identification and measurement (happened in cases # 8 & 10); (iii) a MEP was never detected for unidentified physiological or technical reason (happened in cases # 2-13-14). Heathy controls showed similar issues, but to a lesser extent. Measurable MEPs were obtained in 12/15 and 11/15 controls on the non-dominant and dominant sides, respectively. However, 8 (non-dominant side) and 5 (dominant side) participants were not able to complete TMS testing at 120% rMT because of discomfort (n = 2), large TMS artifact (n = 2) or unidentified physiological/technical reasons (n = 9).

**Table 5:** Results for transcranial magnetic stimulation for each case of SIS.

Case #	RMT (%MSO)		MEP amplitude (*μV*)		MEP latency (ms)	
	Painful	Non/less-painful	Painful	Non/less-painful	Painful	Non/less-painful
**1**	45	60	543.56	1619.55	11.5	15.6
**2**	-	-	-	-	-	-
**3**	59	62	-	-	-	-
**4**	60	63	144.20	-	6.4	-
**5**	83	80	-	-	-	-
**6**	-	69	-	253.36	-	10.3
**7**	-	59	-	340.55	-	11.1
**8**	-	74	-	-	-	-
**9**	52	42	-	-	-	-
**10**	73	-	285.71	-	10.8	-
**11**	56	61	743.41	856.85	13.8	12.5
**12**	79	78	-	-	-	-
**13**	-	-	-	-	-	-
**14**	-	-	-	-	-	-
**15**	60	65	543.56	1619.55	11.8	11.9

SIS = shoulder impingement syndrome; RMT = resting motor threshold; MSO = maximal stimulator output; MEP = motor evoked potential

The proportion of presence/absence of measurable MEPs was compared between sides and groups with the Chi-square test and yielded no significant difference. For subsamples in which MEPs were present, resting motor thresholds were relatively high (~ 60%MSO, Tables 5 and 6), especially in participants with SIS but also in healthy controls. Although rMT values seemed slightly higher for the SIS group (Fig 2B), no significant difference was found between rMT for all within- and between-group comparisons. We decided not to realize statistical comparisons for mean MEP amplitudes and latencies at 120% rMT because of too many lacking data (means and SD or ratio are given in Table 6).

**Table 6:** TMS results of corticospinal excitability for SIS and control groups (mean ± SD or ratio).

	Control group			SIS group		
	Dominant shoulder	Non-dominant shoulder	*Within-group difference (p value)*	Painful shoulder	Non-painful shoulder	*Within-group difference (p value)*
**rMT (% MSO)**	60.45 ± 12.01	56.67 ± 12.01	*0*.*374*	61.84 ± 13.06	65.64 ± 10.25	*0*.*262*
**MEP amplitude (μV)**	621.96 ± 563.91	483.44 ± 574.49	*NT*	434.67 ± 217.27	779.21 ± 617.11	*NT*
**MEP latency (s)**	0.01 ± 0.00	0.01 ± 0.00	*NT*	0.01 ± 0.00	0.01 ± 0.00	*NT*
**Presence / absence of MEP**	11/4	12/3	*0*.*666*	8/7	12/3	*0*.*121 *

N.B. No statistically significant differences for within-group and between-group comparisons; TMS = transcranial magnetic stimulation; SD = standard deviation; SIS = subacromial impingement syndrome; SD = standard deviation; rMT = resting motor treshold; MSO = maximal stimulator output; NT = statistical comparisons were not tested because of lacking data; MEP = motor evoked potential.

## Discussion

The present case series described challenges encountered when using peripheral (VIB) and central (TMS) neurostimulation devices for testing the integrity of sensorimotor processing in individuals with SIS. We also compared their vibration-induced illusions of shoulder movement and shoulder/trunk corticospinal drive between sides and with healthy counterparts. Results only partially support our initial hypotheses. As expected, discomfort and fear-related behaviors were the main challenges reported, especially within the SIS group and for TMS procedures. Furthermore, we hypothesized that illusions would be less clear and of lower speed/amplitude, along with lower corticospinal excitability in the painful shoulder vs. the non-painful one and vs. healthy counterparts. Results showed that processing of sensory information from muscle spindles was mostly spared in the presence of SIS. Similar rate of success, clearness and direction of illusory perceptions were found compared to healthy counterparts, but their speed/amplitude were significantly lower on the affected shoulder compared to the non/less-painful one. Also, neurophysiological measures of cortico-motor control of the upper trapezius muscle failed to detect contrasts of corticospinal excitability between sides and groups. However, too high motor thresholds, TMS-related discomfort and other technical issues precluded from completing TMS measurements, especially in persons with SIS on their painful side. These findings are discussed below, along with recommendations for future clinical and research applications of VIB and TMS tools with populations suffering from musculoskeletal disorders of the shoulder.

### Kinesthetic illusions in shoulder disorders

Two other studies reported tendon vibrations in persons suffering from musculoskeletal shoulder impairments. The 2004 study from Ribot-Ciscar and colleagues applied vibration (80 Hz, 0.5 mm, 15 s duration) on 12 different muscle groups across the upper and lower extremities in 10 healthy and 20 participants with various muscular dystrophies [18]. Overall, no significant difference was found for all tested variables. They did however note a lower (non-significant trend) velocity of illusory perceptions for facioscapulohumeral dystrophy and myotonic dystrophy participants. This observation seems to agree with our finding of a lower illusory speed/amplitude on the painful side in SIS group. The 2015 study from Ribot-Ciscar et al. applied vibratory stimuli to shoulder and arm muscles, including the triceps and biceps brachii as well as the pectoralis major, respectively for inducing illusions of elbow flexion, extension and shoulder abduction. In addition to reporting the perceived sensations in eight persons with facioscapulohumeral dystrophy, they provided eight 40–60 min sessions of vibratory treatment (named vibratory proprioceptive assistance) for a month. The therapeutic protocol included six blocks of stimulation eliciting a mix of uniplanar and multiplanar elbow/shoulder movements by stimulating the three targeted muscles alone or in combination. Their results showed that participants were able to perceive clear illusions in the expected direction coherent with the role of the stimulated muscles spindles [43] like we found with the SIS population. Interestingly, participants reported that the amplitude of illusory perceptions often exceeded what they were able to reach voluntarily. The experimenters in 2015 Ribot-Ciscar’s study also frequently reported antagonistic vibratory responses during kinesthetic illusions, i.e. involuntary contractions of muscles antagonistic to those vibrated [17]. These motor responses are likely highlighting vibration-induced facilitation of sensorimotor control networks coherent with the perceived movement [13, 39]. Kinesthetic illusions are known to activate key fronto-parietal and cerebellar networks involved in voluntary motor control [20, 44]. In accordance with these observations, the vibratory proprioceptive assistance protocol led to significant improvements in shoulder ranges of motion, isometric strength, self-rated shoulder function and health [17]. The repeated and painless perception of shoulder/elbow movements in patients with sensorimotor dysfunctions could have induced long-lasting plastic changes within these neural networks [17]. Considering the available literature, our results are mostly encouraging further research testing the effectiveness of VIB-based treatments in persons with SIS. Their relatively intact processing of VIB-induced afferents, like found with fascioscapulohumeral dystrophic populations, could suggest a potential for positive plastic changes within shoulder sensorimotor control networks. Since the present work used VIB-induced illusions to verify the integrity of proprioception processing and not as a treatment, further research is needed to test the effects of repetitive VIB-induced illusions with SIS groups.

The lower speed/amplitude of illusions obtained on the painful side in our study might be implying a less effective, but still functional processing of muscle spindles afferents. Pain and non-use of affected limbs can have a profound impact on neural networks involved in perceptual, cognitive/emotional and motor functions [45–48]. On the other hand, individual data (Fig 2A) shows that this lower speed/amplitude was not systematic. As seen in Table 3, 2 participants perceived a faster speed/amplitude on their painful side compared to their non/less painful one (cases #7 and #12), and 4 individuals (cases# 2-3-6-14) had relatively similar scores within a 1–3.7% range. Healthy controls also had important between and within-subject variations of speed/amplitude (Fig 2A). Other sources of variation such as higher difficulty to fully relax during VIB, for example with case #12 who showed fear of pain behaviors, could also explain the lower speed/amplitude perceived. Complete relaxation is an important factor to control for eliciting kinesthetic illusions [17, 30]. To minimize this risk, care was taken before each VIB trial to ensure the subject’s relaxation (gently moving the arm, asking the participant to relax, etc.), following recommendations from the Standardized Kinesthetic Illusion Procedure [30]. Future studies might consider using biofeedback methods (e.g. electromyography) to verify the level of relaxation before applying VIB in populations at higher risks of presenting kinesiophobic and protective attitudes. High intra and inter-individual variability of speed/amplitude could also underscore that illusory perceptions present a natural variation from trial to trial and between persons. The metrological properties of the different methods used to measure kinesthetic illusions remain to be established–a low reliability for instance could explain the high variability observed.

### Corticospinal excitability in shoulder disorders

A recent study [24] tested corticospinal excitability of the affected upper trapezius, lower trapezius and serratus anterior muscles in 14 persons with SIS. All TMS procedures were realized in an active muscle state, i.e. participants had to actively maintain a 90° scapular plan elevation. Compared to a resting state, an active contraction of the target muscle is known to reduce the threshold for eliciting MEPs, through a higher state of excitability from the involved pool of interneurons and motoneurons located at M1 and spinal levels [36]. The authors stated in their methods that the active state was chosen because they found it difficult to elicit MEPs when the targeted muscles were at rest. From a methodological standpoint, our results give weight to this observation, since TMS measures were challenged by high motor thresholds and sometimes intolerable stimuli intensities. Furthermore, Lin and coworkers tested infraspinatus and deltoid muscles on 17 healthy participants and found that changing the arm position from 0 to 90° of elevation increased the maximal MEP value for the infraspinatus, but not the deltoid, highlighting the importance of also considering the arm position when testing cortico-motor functions of shoulder muscles with TMS [49]. However, from a patient-centered point of view, at least based on our recruited sample of persons with SIS, maintaining a 90° shoulder elevation would have affected their capacity and motivation to complete TMS testing. Fatigue and pain build-up can directly affect the validity and reliability of TMS outcome measures [50–52]. In our study, even if the participant’s arm was resting for the most part of the two experimental sessions and that the duration of each session was kept below 1.5 hours, persons with SIS often asked for small breaks. As described in the results, significant discomfort and fear of pain behaviors were reported during vibration and TMS, which often required reassurance that these techniques were safe. According to Chung et al.’s (2022), none of their participants with SIS complained of pain during their 3–4 hours experiment. Differences in our respective protocols and, more importantly, characteristics of participants could explain this discrepancy. Nevertheless, our results raise awareness on challenges inherent to using TMS in populations affected by musculoskeletal pain and disabilities.

Despite our missing TMS data in several participants, we were still able to compare motor thresholds and success rate to elicit MEPs. No significant difference for inter and intra-group comparisons was found. As shown in Tables 5 and 6, thresholds near or above 60% were quite frequent but not restricted to SIS participants. Conversely, Chung and colleagues (2022) reported a decreased corticospinal excitability (higher motor thresholds) of lower trapezius and serratus anterior, higher cortico-cortical and spinal inhibitory mechanisms (longer silent period) for the lower trapezius and cortical reorganization of M1 motor maps (posterior shift of the centre of gravity) in the upper trapezius and serratus anterior. Berth et al. tested the deltoid muscle and first interosseus muscle on 10 participants with chronic rotator cuff tears and 13 healthy controls. They found a higher corticospinal excitability for the rotator cuff tears group when the tested arm remained at rest but a lower excitability was observed when they had to maintain a 5–10% maximal isometric contraction [23]. Ngomo and colleagues found higher motor thresholds (lower excitability) for the infraspinatus (active state of about 5% of maximal isometric contraction) in a group of 39 persons with rotator cuff tendinopathy when comparing with the unaffected side. This finding was correlated with the duration of symptoms, but not their intensity [46]. In 2007, Alexander compared corticospinal excitability of the upper and lower trapezius between 11 persons with non-traumatic shoulder-instability and 8 healthy controls. They found similar corticospinal excitability between groups for the upper trapezius but lower excitability for the lower trapezius muscle [53]. Overall, our preliminary results are not agreeing with other publications in the field. We found no clear sign of an altered corticospinal function in the presence of shoulder impairments. As seen in Tables 5 and 6, rMT varied greatly both within and between groups. However, comparisons between studies are hindered by high heterogeneity of methods and populations, and the strength of our results can be questioned based on missing TMS data (except for MEP presence/absence). Nevertheless, our study highlights the fact that pairing VIB and TMS technologies appear complementary for a specific investigation of mechanisms underlying sensory (VIB) and motor (TMS) impairments. A recent study from our group delivered TMS at different time points *during* VIB-induced illusion in healthy persons and observed a time-specific modulation of corticospinal drive, which was lowest after VIB start compared to later when the illusion had time to build up [54]. The pairing of VIB and TMS with this new testing paradigm could help affine the evaluation of sensorimotor processing mechanisms and their integrity in SIS populations.

## Limitations

Results from this research is limited because of the small sample size and loss of TMS data. An *a priori* sample size to ensure at least 95% of power and alpha risk below 5% was calculated and recommended at least 13 participants per group for VIB-related outcomes and 15 per group for TMS measures obtained with the upper trapezius muscle. This power-based recommendation was respected for VIB outcomes, but not for TMS. Further studies with higher statistical power are highly needed for improving our understanding of VIB and TMS-related mechanisms of sensorimotor control, and possibly their effects if used as therapeutic protocols.

Our results also call for further research identifying the best VIB and TMS measurement methods adapted to the needs and challenges of populations suffering from musculoskeletal pain and disabilities. Discomfort, kinesiophobia and fatigue should be closely monitored during the experiment, for example using visual analog scales or the Borg questionnaire of perceived exertion [55]. Also, stimulating M1 representations of trunk or proximal muscles like the upper trapezius could be facilitated by using different coil sizes and shape, like the double cone coil used for reaching the deeper representation of leg muscles [56]. One unexpected challenge we encountered was the large TMS-induced artifacts that prevented MEP identification in EMG data in a few participants. This never occurred in our previous studies targeting more distal upper and lower limb muscles. There are two possible explanations for this technical challenge. First, our EMG system does not realize a pre-amplification of the signal at the level of the recording electrodes. Instead, the signal from the two active electrodes is sent through cables toward the CED 1902 amplifier. A common-mode rejection is then applied using the signal from the ground electrode to reduce electrical inputs from sources other than the muscles. The high TMS intensities and proximity of the electrodes from the coil probably explain why our system failed to identify and remove the TMS artifact from EMG signals. Different methods should be tested in future work to identify the best way of amplifying/filtering EMG signals when targeting proximal trunk/upper-limb muscles (e.g. pre-amplification, input clamps, location of the ground). Another potential reason for this technical issue was EMG location. We strictly respected SENIAM guidelines for electrode placement but observed that the resulting location was sometimes not above the biggest part of the muscle belly. A sub-optimal EMG location could have resulted in weaker electrical inputs, which would have in turn affected MEP detection, increased motor thresholds, and reduced the efficacy of the common-mode rejection. Future studies should also compare different EMG location methods as it could directly impact the feasibility and validity of TMS.

Interestingly, physiatrist’ (MBR) reports based on ultrasound imagery identified structural damage in both the painful (66.7% of cases) and non/less-painful sides (53.3% of cases) of persons with SIS. Mild damages to subacromial structures were even found in at least one shoulder in 60% of healthy participants. Asymptomatic damage to subacromial structures could have affected comparisons between SIS and ‘healthy’ controls. Or, alternatively, non-symptomatic shoulders simply present normal age- & use-related lesions that form without any pain or dysfunction [57, 58]. This agrees with previous reports having questioned the mechanical impingement and resulting subacromial lesions as the primary mechanism leading to SIS [2, 59–65].

## Conclusions

Our study highlighted several challenges related to the use of central and peripheral neurostimulation devices with people having shoulder pain. In particular, TMS procedures were affected by technical problems, high intensities and related discomfort that was often exacerbated by a fear of pain in persons with SIS. Our experience will hopefully help future research to better address those challenges and improve the feasibility of VIB and TMS. It also provided novel evidence on the integrity of proprioceptive and corticospinal functions in persons living with shoulder impingement syndrome. Compared to healthy counterparts, they showed a similar capacity to perceive kinesthetic illusions on their affected shoulder, which were in the expected direction of shoulder abduction. They were however of lower speed/amplitude when comparing sides in the SIS group. Results failed to detect a difference of corticospinal excitability between groups and sides and encourage future work to determine the best methodological procedures for increasing the relevance and success-rate of TMS when testing populations suffering from musculoskeletal pain and disabilities. Nevertheless, neurostimulation tools like VIB and TMS show promise to better understand mechanisms underlying sensorimotor dysfunctions in those populations, and even propose novel therapeutic methods that could specifically target these mechanisms.

## References

[1] Luime JJ, Koes BW, Hendriksen IJ, Burdorf A, Verhagen AP, Miedema HS, et al. Prevalence and incidence of shoulder pain in the general population; a systematic review. Scand J Rheumatol. 2004;33(2):73–81. doi: 10.1080/03009740310004667 . 15163107

[2] Dhillon K. Subacromial impingement syndrome of the shoulder: a musculoskeletal disorder or a medical myth? Malaysian orthopaedic journal. 2019;13(3):1. doi: 10.5704/MOJ.1911.001 31890103 PMC6915323

[3] Lewis JS, Wright C, Green A. Subacromial impingement syndrome: the effect of changing posture on shoulder range of movement. The Journal of orthopaedic and sports physical therapy. 2005;35(2):72–87. Epub 2005/03/19. doi: 10.2519/jospt.2005.35.2.72 . 15773565

[4] Umer M, Qadir I, Azam M. Subacromial impingement syndrome. Orthopedic reviews. 2012;4(2): e18. doi: 10.4081/or.2012.e18 22802986 PMC3395987

[5] Lopes AD, Timmons MK, Grover M, Ciconelli RM, Michener LA. Visual scapular dyskinesis: kinematics and muscle activity alterations in patients with subacromial impingement syndrome. Arch Phys Med Rehabil. 2015;96(2):298–306. Epub 2014/12/03. doi: 10.1016/j.apmr.2014.09.029 . 25449194

[6] Struyf F, Nijs J, De Graeve J, Mottram S, Meeusen R. Scapular positioning in overhead athletes with and without shoulder pain: a case-control study. Scandinavian Journal of Medicine & Science in Sports. 2011;21(6):809–18. doi: 10.1111/j.1600-0838.2010.01115.x 20500559

[7] Anderson VB, Wee E. Impaired joint proprioception at higher shoulder elevations in chronic rotator cuff pathology. Archives of physical medicine and rehabilitation. 2011;92(7):1146–51. doi: 10.1016/j.apmr.2011.02.004 21704796

[8] Ager AL, Borms D, Deschepper L, Dhooghe R, Dijkhuis J, Roy JS, et al. Proprioception: How is it affected by shoulder pain? A systematic review. J Hand Ther. 2020;33(4):507–16. Epub 2019/09/05. doi: 10.1016/j.jht.2019.06.002 . 31481340

[9] Ager AL, Borms D, Bernaert M, Brusselle V, Claessens M, Roy JS, et al. Can a Conservative Rehabilitation Strategy Improve Shoulder Proprioception? A Systematic Review. J Sport Rehabil. 2020:1–16. Epub 2020/08/01. doi: 10.1123/jsr.2019-0400 . 32736342

[10] Warner JJ, Lephart S, Fu FH. Role of proprioception in pathoetiology of shoulder instability. Clinical Orthopaedics and Related Research®. 1996;330:35–9. doi: 10.1097/00003086-199609000-00005 8804272

[11] Roll JP, Vedel JP, Ribot E. Alteration of proprioceptive messages induced by tendon vibration in man: a microneurographic study. Experimental brain research. 1989;76(1):213–22. doi: 10.1007/BF00253639 2753103

[12] Roll JP, Albert F, Thyrion C, Ribot-Ciscar E, Bergenheim M, Mattei B. Inducing any virtual two-dimensional movement in humans by applying muscle tendon vibration. Journal of neurophysiology. 2009;101(2):816–23. Epub 2008/12/05. doi: 10.1152/jn.91075.2008 . 19052107

[13] Calvin-Figuière S, Romaiguère P, Gilhodes JC, Roll JP. Antagonist motor responses correlate with kinesthetic illusions induced by tendon vibration. Experimental brain research. 1999;124(3):342–50. doi: 10.1007/s002210050631 9989440

[14] Thyrion C, Roll J-P. Predicting any arm movement feedback to induce three-dimensional illusory movements in humans. Journal of neurophysiology. 2010;104(2):949–59. doi: 10.1152/jn.00025.2010 20538782

[15] Conrad MO, Scheidt RA, Schmit BD. Effects of wrist tendon vibration on arm tracking in people poststroke. Journal of Neurophysiology. 2011;106(3):1480–8. doi: 10.1152/jn.00404.2010 21697444 PMC3174817

[16] Beaulieu L-D, Schneider C, Ribot-Ciscar E. Interventions non invasives en phase chronique post-AVC: rôle des afférences proprioceptives sur la plasticité cérébrale et le contrôle sensorimoteur. Québec: Université Laval; 2016.

[17] Ribot-Ciscar E, Milhe-De Bovis V, Aimonetti J-M, Lapeyssonnie B, Campana-Salort E, Pouget J, et al. Functional impact of vibratory proprioceptive assistance in patients with facioscapulohumeral muscular dystrophy. Muscle & nerve. 2015;52(5):780–7. doi: 10.1002/mus.24605 25678042

[18] Ribot-Ciscar E, Tréfouret S, Aimonetti J-M, Attarian S, Pouget J, Roll J-P. Is muscle spindle proprioceptive function spared in muscular dystrophies? A muscle tendon vibration study. Muscle & Nerve. 2004;29(6):861–6. doi: 10.1002/mus.20044 15170619

[19] Naito E, Morita T, Amemiya K. Body representations in the human brain revealed by kinesthetic illusions and their essential contributions to motor control and corporeal awareness. Neuroscience Research. 2016;104:16–30. doi: 10.1016/j.neures.2015.10.013 26562333

[20] Romaiguère P, Anton J-L, Roth M, Casini L, Roll J-P. Motor and parietal cortical areas both underlie kinaesthesia. Cognitive Brain Research. 2003;16(1):74–82. doi: 10.1016/s0926-6410(02)00221-5 12589891

[21] O’Sullivan SB, Schmitz TJ, Fulk G. Physical rehabilitation. F.A. Davis company, Philadelphia: FA Davis; 2019.

[22] Chen R, Cros D, Curra A, Di Lazzaro V, Lefaucheur JP, Magistris MR, et al. The clinical diagnostic utility of transcranial magnetic stimulation: report of an IFCN committee. Clin Neurophysiol. 2008;119(3):504–32. Epub 2007/12/08. S1388-2457(07)00618-9 [pii] doi: 10.1016/j.clinph.2007.10.014 . 18063409

[23] Berth A, Pap G, Neuman W, Awiszus F. Central neuromuscular dysfunction of the deltoid muscle in patients with chronic rotator cuff tears. J Orthop Traumatol. 2009;10(3):135–41. Epub 2009/08/20. doi: 10.1007/s10195-009-0061-7 ; PubMed Central PMCID: PMC2744738. 19690944 PMC2744738

[24] Chung YC, Chen CY, Chang CM, Lin YL, Liao KK, Lin HC, et al. Altered corticospinal excitability of scapular muscles in individuals with shoulder impingement syndrome. PLoS One. 2022;17(5): e0268533. Epub 20220516. doi: 10.1371/journal.pone.0268533 ; PubMed Central PMCID: PMC9109916. 35576229 PMC9109916

[25] Faul F, Erdfelder E, Lang AG, Buchner A. G*Power 3: a flexible statistical power analysis program for the social, behavioral, and biomedical sciences. Behav Res Methods. 2007;39(2):175–91. doi: 10.3758/bf03193146 . 17695343

[26] Michener LA, Walsworth MK, Doukas WC, Murphy KP. Reliability and diagnostic accuracy of 5 physical examination tests and combination of tests for subacromial impingement. Archives of physical medicine and rehabilitation. 2009;90(11):1898–903. Epub 2009/11/06. doi: 10.1016/j.apmr.2009.05.015 . 19887215

[27] Rossi S, Hallett M, Rossini PM, Pascual-Leone A, Safety of TMSCG. Safety, ethical considerations, and application guidelines for the use of transcranial magnetic stimulation in clinical practice and research. Clinical neurophysiology: official journal of the International Federation of Clinical Neurophysiology. 2009;120(12):2008–39. doi: 10.1016/j.clinph.2009.08.016 19833552 PMC3260536

[28] Durand MJ, Vachon B, Hong QN, Loisel P. The cross-cultural adaptation of the DASH questionnaire in Canadian French. Journal of hand therapy: official journal of the American Society of Hand Therapists. 2005;18(1):34–9. Epub 2005/01/28. doi: 10.1197/j.jht.2004.10.010 . 15674785

[29] Miller R, Doyle S, Arotca A. Reliability of a US version of the Nottingham sensory assessment. The American Journal of Occupational Therapy. 2016;70(4_Supplement_1):7011500015p1–p1.

[30] Beaulieu L-D, Schneider C, Massé-Alarie H, Ribot-Ciscar E. A new method to elicit and measure movement illusions in stroke by means of muscle tendon vibration: the Standardized Kinesthetic Illusion Procedure (SKIP). Somatosensory & motor research. 2020;37(1):28–36. doi: 10.1080/08990220.2020.1713739 31973656

[31] Scott J, Huskisson E. Vertical or horizontal visual analogue scales. Annals of the rheumatic diseases. 1979;38(6):560. doi: 10.1136/ard.38.6.560 317239 PMC1000420

[32] Hermens HJ, Freriks B, Disselhorst-Klug C, Rau G. Development of recommendations for SEMG sensors and sensor placement procedures. Journal of electromyography and Kinesiology. 2000;10(5):361–74. doi: 10.1016/s1050-6411(00)00027-4 11018445

[33] Klem GH. The ten-twenty electrode system of the international federation. the internanional federation of clinical nenrophysiology. Electroencephalogr Clin Neurophysiol Suppl. 1999;52:3–6. 10590970

[34] Herwig U, Satrapi P, Schönfeldt-Lecuona C. Using the international 10–20 EEG system for positioning of transcranial magnetic stimulation. Brain topography. 2003;16(2):95–9. doi: 10.1023/b:brat.0000006333.93597.9d 14977202

[35] Sakai K, Ugawa Y, Terao Y, Hanajima R, Furubayashi T, Kanazawa I. Preferential activation of different I waves by transcranial magnetic stimulation with a figure-of-eight-shaped coil. Experimental Brain Research. 1997;113(1):24–32. doi: 10.1007/BF02454139 9028772

[36] Rossini PM, Burke D, Chen R, Cohen L, Daskalakis Z, Di Iorio R, et al. Non-invasive electrical and magnetic stimulation of the brain, spinal cord, roots and peripheral nerves: Basic principles and procedures for routine clinical and research application. An updated report from an IFCN Committee. Clinical neurophysiology. 2015;126(6):1071–107. 25797650 10.1016/j.clinph.2015.02.001 PMC6350257

[37] Awiszus F. TMS and threshold hunting. Supplements to Clinical neurophysiology. 56: Elsevier; 2003. p. 13–23. 14677378 10.1016/s1567-424x(09)70205-3

[38] Rossini PM, Barker A, Berardelli A, Caramia M, Caruso G, Cracco R, et al. Non-invasive electrical and magnetic stimulation of the brain, spinal cord and roots: basic principles and procedures for routine clinical application. Report of an IFCN committee. Electroencephalography and clinical neurophysiology. 1994;91(2):79–92. doi: 10.1016/0013-4694(94)90029-9 7519144

[39] Calvin-Figuière S, Romaiguère P, Roll JP. Relations between the directions of vibration-induced kinesthetic illusions and the pattern of activation of antagonist muscles. Brain research. 2000;881(2):128–38. doi: 10.1016/s0006-8993(00)02604-4 11036150

[40] Le Franc S, Bonan I, Fleury M, Butet S, Barillot C, Lécuyer A, et al. Visual feedback improves movement illusions induced by tendon vibration after chronic stroke. Journal of neuroengineering and rehabilitation. 2021;18(1):1–9. 34717672 10.1186/s12984-021-00948-7 PMC8556973

[41] Goble DJ, Coxon JP, Van Impe A, Geurts M, Doumas M, Wenderoth N, et al. Brain activity during ankle proprioceptive stimulation predicts balance performance in young and older adults. Journal of Neuroscience. 2011;31(45):16344–52. doi: 10.1523/JNEUROSCI.4159-11.2011 22072686 PMC6633212

[42] Goble DJ, Coxon JP, Van Impe A, Geurts M, Van Hecke W, Sunaert S, et al. The neural basis of central proprioceptive processing in older versus younger adults: an important sensory role for right putamen. Human brain mapping. 2012;33(4):895–908. doi: 10.1002/hbm.21257 21432946 PMC6870471

[43] Roll JP, Vedel JP. Kinaesthetic role of muscle afferents in man, studied by tendon vibration and microneurography. Experimental brain research. 1982;47(2):177–90. doi: 10.1007/BF00239377 6214420

[44] Naito E, Nakashima T, Kito T, Aramaki Y, Okada T, Sadato N. Human limb‐specific and non‐limb‐specific brain representations during kinesthetic illusory movements of the upper and lower extremities. European Journal of Neuroscience. 2007;25(11):3476–87. doi: 10.1111/j.1460-9568.2007.05587.x 17553017

[45] Luchtmann M, Firsching R. Central plasticity resulting from chronic low back pain in degenerative disorders of the spine. Neural Regeneration Research. 2015;10(8):1234. doi: 10.4103/1673-5374.162754 26487848 PMC4590233

[46] Ngomo S, Mercier C, Bouyer LJ, Savoie A, Roy JS. Alterations in central motor representation increase over time in individuals with rotator cuff tendinopathy. Clinical neurophysiology: official journal of the International Federation of Clinical Neurophysiology. 2015;126(2):365–71. Epub 2014/07/22. doi: 10.1016/j.clinph.2014.05.035 . 25043198

[47] Kleim JA, Jones TA. Principles of Experience-Dependent Neural Plasticity: Implications for Rehabilitation after Brain Damage. Journal of Speech, Language, and Hearing Research. 2008;51: S225–S39. 10.1044/1092-4388(2008/018) 18230848

[48] Langer N, Hänggi J, Müller NA, Simmen HP, Jäncke L. Effects of limb immobilization on brain plasticity. Neurology. 2012;78(3):182–8. doi: 10.1212/WNL.0b013e31823fcd9c 22249495

[49] Lin YL, Christie A, Karduna A. Excitability of the infraspinatus, but not the middle deltoid, is affected by shoulder elevation angle. Exp Brain Res. 2015;233(6):1837–43. Epub 2015/03/31. doi: 10.1007/s00221-015-4255-3 . 25814379

[50] Farina S, Valeriani M, Rosso T, Aglioti S, Tamburin S, Fiaschi A, et al. Transient inhibition of the human motor cortex by capsaicin-induced pain. A study with transcranial magnetic stimulation. Neuroscience letters. 2001;314(1–2):97–101. doi: 10.1016/s0304-3940(01)02297-2 11698155

[51] Rittig-Rasmussen B, Kasch H, Fuglsang-Frederiksen A, Svensson P, Jensen TS. The role of neuroplasticity in experimental neck pain: a study of potential mechanisms impeding clinical outcomes of training. Manual Therapy. 2014;19(4):288–93. doi: 10.1016/j.math.2014.04.010 24815594

[52] Taylor JL, Gandevia SC. Transcranial magnetic stimulation and human muscle fatigue. Muscle Nerve. 2001;24(1):18–29. Epub 2001/01/11. doi: 10.1002/1097-4598(200101)24:1&lt;18::aid-mus2&gt;3.0.co;2-d . 11150962

[53] Alexander CM. Altered control of the trapezius muscle in subjects with non-traumatic shoulder instability. Clin Neurophysiol. 2007;118(12):2664–71. Epub 2007/10/24. doi: 10.1016/j.clinph.2007.09.057 . 17950033

[54] Lauzier L, Perron MP, Munger L, Bouchard E, Abboud J, Nougarou F, et al. Variation of corticospinal excitability during kinesthetic illusion induced by musculotendinous vibration. J Neurophysiol. 2023;130(5):1118–25. Epub 20230914. doi: 10.1152/jn.00069.2023 . 37706230

[55] Borg G. Borg’s perceived exertion and pain scales. PsycInfo Database Record: Human kinetics; 1998.

[56] Davies JL. Using transcranial magnetic stimulation to map the cortical representation of lower-limb muscles. Clinical Neurophysiology Practice. 2020;5:87–99. doi: 10.1016/j.cnp.2020.04.001 32455179 PMC7235616

[57] Sher JS, Uribe JW, Posada A, Murphy BJ, Zlatkin MB. Abnormal findings on magnetic resonance images of asymptomatic shoulders. The Journal of bone and joint surgery American volume. 1995;77(1):10–5. doi: 10.2106/00004623-199501000-00002 7822341

[58] Schwartzberg R, Reuss BL, Burkhart BG, Butterfield M, Wu JY, McLean KW. High prevalence of superior labral tears diagnosed by MRI in middle-aged patients with asymptomatic shoulders. Orthopaedic journal of sports medicine. 2016;4(1):2325967115623212. doi: 10.1177/2325967115623212 26779556 PMC4710128

[59] Dorrestijn O, Stevens M, Winters JC, van der Meer K, Diercks RL. Conservative or surgical treatment for subacromial impingement syndrome? A systematic review. Journal of shoulder and elbow surgery. 2009;18(4):652–60. doi: 10.1016/j.jse.2009.01.010 19286397

[60] Holmgren T, Hallgren HB, Öberg B, Adolfsson L, Johansson K. Effect of specific exercise strategy on need for surgery in patients with subacromial impingement syndrome: randomised controlled study. Bmj. 2012;344. doi: 10.1136/bmj.e787 22349588 PMC3282676

[61] Khan M, Alolabi B, Horner N, Bedi A, Ayeni OR, Bhandari M. Surgery for shoulder impingement: a systematic review and meta-analysis of controlled clinical trials. Canadian Medical Association Open Access Journal. 2019;7(1): E149–E58. doi: 10.9778/cmajo.20180179 30846616 PMC6411477

[62] Requejo-Salinas N, Lewis J, Michener LA, La Touche R, Fernández-Matías R, Tercero-Lucas J, et al. International physical therapists consensus on clinical descriptors for diagnosing rotator cuff related shoulder pain: A Delphi study. Brazilian journal of physical therapy. 2022;26(2):100395. doi: 10.1016/j.bjpt.2022.100395 35366589 PMC8978275

[63] Lewis JS. Rotator cuff tendinopathy/subacromial impingement syndrome: is it time for a new method of assessment? British journal of sports medicine. 2009;43(4):259–64. Epub 2008/10/08. doi: 10.1136/bjsm.2008.052183 . 18838403

[64] Lewis J. Rotator cuff related shoulder pain: Assessment, management and uncertainties. Manual Therapy. 2016;23:57–68. doi: 10.1016/j.math.2016.03.009 27083390

[65] McFarland EG, Maffulli N, Del Buono A, Murrell GA, Garzon-Muvdi J, Petersen SA. Impingement is not impingement: the case for calling it “Rotator Cuff Disease”. Muscles, ligaments and tendons journal. 2013;3(3):196. 24367779 PMC3838328

